# Review of Levetiracetam as a First Line Treatment in Status Epilepticus in the Adult Patients – What Do We Know so Far?

**DOI:** 10.3389/fneur.2013.00111

**Published:** 2013-08-05

**Authors:** Hae Won Shin, Robin Davis

**Affiliations:** ^1^Department of Neurology, University of North Carolina Health Care, Chapel Hill, NC, USA

**Keywords:** levetiracetam, status epilepticus, seizure, first-line therapy for the status epilepticus, antiepileptic drug

## Abstract

With the advent of new antiepileptic drugs comes the potential for significant advances in the emergent management of status epilepticus. Traditional antiepileptic drugs possess side effect profiles that may limit their clinical utility or lead to increased patient morbidity or mortality. The relatively recent development of levetiracetam shows promise for effective control of acute status epilepticus in adults, but current objective data of its use as a first-line agent for control of status is quite limited. This paper serves to examine existing literature while considering levetiracetam as a first-line therapy in status in the adult patient population. Although existing studies are narrow in their scope, the present data lay a substantial foundation for further investigation of levetiracetam as a primary therapy in acute status epilepticus.

## Introduction

The emergent management of status epilepticus has been restricted to a small subset of antiepileptic drugs suitable for acute intravenous infusion. Of the three major traditional drugs used in first-line therapy for status, each carries a side effect profile that may ultimately lend to limited clinical utility in the critical patient. Lorazepam, phenytoin, and phenobarbital individually convey specific risks to the patient, among them respiratory depression, medication interaction due to cyp450 induction, and teratogenicity (1). Each of these known effects may complicate their use in an urgent setting and contribute to increased morbidity in patients presenting with status epilepticus (1).

Given its minimal known side effect profile, limited drug interactions, and availability as a rapid delivery IV formulation, levetiracetam, a relatively new AED by comparison, may be a viable, and practical option in the first-line management of status epilepticus (2, 3). Status epilepticus has been defined most commonly as seizure activity persisting for over 30 min, inclusive of tonic, absence, complex partial, convulsive, non-convulsive, and myoclonic seizure types, among others. The majority of data regarding the use of levetiracetam in status epilepticus has been applied to generalized seizure types, though there is growing interest in its application for a variety of epileptic activity (4).

## Traditional and Emerging Management of Status Epilepticus

The traditional approach taken in the emergent management of status was described by Riviello et al. (5) in their survey of 120 physicians who were asked to manage hypothetical patients diagnosed with status epilepticus (5). When given the case of an adult male without a known underlying seizure disorder, the physician subjects overwhelmingly initiated benzodiazepine therapy first, choosing lorazepam followed by phenytoin as a secondary agent (5). Levetiracetam was not chosen until the physician was required to select a third agent, with it being selected with the same frequency as midazolam, propofol, or phenobarbital (5). Cook et al. (6) echoed this sentiment with a 150 patient retrospective study, again noting that in real clinical applications, physicians readily followed the first-line benzodiazepine trailed by phenytoin model (6). Although levetiracetam was not chosen until the patient required a third-line agent, Cook et al. (6) did note that 10% of patients received levetiracetam as a second-line agent in their evaluations (6). Additionally, levetiracetam was the second most-selected third-line agent. The authors postulate that this may be indicative of growing acceptance of levetiracetam as a promising therapy in status (6). Cook et al. (6) did call attention to 65.1% of patients in the study continuing levetiracetam therapy upon discharge home with a particular frequency in patients with no prior history of seizure (6). Taken together, this is suggestive of a slowly developing physician comfort with levetiracetam.

The reasoning behind the traditional management of status is perhaps most apparent in Brophy et al.’s (7) discussion of the Neurocritical Care Society’s Status Epilepticus Guideline Writing Committee, which set forth parameters for a stepwise approach to the management of status epilepticus (7). This initial set of standards favored benzodiazepine therapy as a first-line modality citing the utility of the multiple administration routes and rapid infusion times common to many benzodiazepines, most notably lorazepam, and midazolam (7). The guidelines do offer the caveat of respiratory depression with benzodiazepine therapy with the potential need for intubation with repeated dosing (7). While the committee limited their recommendations for the emergent control of status epilepticus to benzodiazepines, levetiracetam was given a strong recommendation for urgent control of status along with two other drugs, valproate sodium and phenytoin/fosphenytoin (7). Of these drugs, however, levetiracetam is the only agent not noted in the guidelines for having a serious potential side effect profile (7). Although levetiracetam currently is listed as having only Class IIA, Level C evidence supporting its use in emergent, urgent, and refractory status, its adoption as a strongly recommended therapy for urgent status epilepticus provides for reasonable interpretation that an expansion of available clinical evidence could lead to its eventual acceptance as a first-line therapy in the emergent management of status (7).

## Levetiracetam as a First-Line Agent

While levetiracetam is approved as an adjuvant agent for the management of status, data on its use as a first-line therapy is quite limited, as noted in the Neurocritical Care Society’s Guidelines discussed previously. Review of available literature via PubMed search reveals fewer than 10 reports of levetiracetam used as a first-line agent in status, with only a single randomized pilot study and a limited number of case and retrospective reports available in current publication. Despite this, evaluating existing data as a whole opens the discussion for levetiracetam administration as a first-line approach in status epilepticus.

A clinical pilot performed by Misra et al. (8) provides the only direct comparison of levetiracetam and a traditional first-line agent, lorazepam, in available literature (8). A loading dose of lorazepam or levetiracetam was administered to each of 79 patients between the ages of 1 and 75 with convulsive or subtle convulsive status epilepticus (defined as two or more seizures) (8). While pediatric patients were included in the study, no formal breakdown of the age distribution of the patient population was included in the study report. It should be noted that the mean age of subjects receiving lorazepam was 38.9 years with a mean age of 39.2 for patients receiving levetiracetam. Levetiracetam infusion controlled status in 76.3% of patients with lorazepam achieving the same in 75.6% (8). Slightly improved 24-h seizure freedom was noted in patients in the levetiracetam group (8). Interestingly, there was more frequent need for artificial ventilation in patients receiving first-line lorazepam, though no significant difference in mortality between the two groups was ultimately illustrated (8). While this pilot was hindered by limited power due to subject recruiting, it stands as the only available direct comparison between a traditional first-line agent and levetiracetam (8).

In a review written by Zelano and Kumlien (9), levetiracetam was evaluated as a stage two therapy in status management in an analysis of 10 studies (9). The review included three prospective and seven retrospective studies, which comprised total 334 patients (9). Ultimately, the studies represented an efficacy range for levetiracetam of 44–94% (9). The authors’ work further substantiates the notion of levetiracetam as an effective clinical option with applications to a broader range of patients, not just those without potential drug interactions, feared toxicity, or other foreseen adverse reactions to traditional therapies.

## Utility in Pharmacology and Tolerability

Levetiracetam’s limited side effect profile makes it particularly appealing in older patient populations, where drug interaction and the potential for intubation leading to significant mortality is of great concern. Fattouch et al. explored this in a limited retrospective of nine patients, older than 65 years old with video EEG confirmed status epilepticus who received loading doses of 1500 mg IV levetiracetam as a first-line agent (10). Eight of nine cases responded to loading dose within 15–30 min of dosing with confirmation obtained via EEG, with only one of whom experienced a few isolated seizures in the days following dosing (10).

Additionally, Farooq et al. (11) reported two cases of patients, older than 80 years old who responded within 35 min to levetiracetam load (11). This limited report features the cases of a patient already receiving daily levetiracetam therapy who had missed a dose and another patient found to be therapeutic on phenytoin whose seizures ceased within a minute of levetiracetam administration (11).

Berning et al. (12) addressed the issue of first-line levetiracetam in patients in whom there was a high level of concern for the risks of coma induction and subsequent increased mortality (12). The group reported on 2 patients out of a larger group of 32 individuals in status who were treated exclusively with levetiracetam within 6 h of seizure onset and who responded to therapy between 6 and 18 h after administration of the agent and required no further therapy (12). Berning et al. (12) also incorporated patients categorized as “low first line therapy,” specifically eight patients treated with low dose (2 mg) lorazepam or equivalent prior to administration of levetiracetam in lieu of “high first line” or “traditional first line therapies” (12). In this case, the patients who received either no traditional first-line drugs or only low dose benzodiazepines had lower change in morbidity when compared to more aggressive traditional first-line interventions (12).

## Levetiracetam in the Critically Ill

It is the potential for limited increase in mortality that makes levetiracetam particularly appealing for first-line use in critically ill patients, which was explored by Rüegg et al. (13). In their analysis, 12 patients received first-line levetiracetam for status epilepticus or seizure as part of a larger 50 patient study (13). Eleven of 12 patients became and remained seizure free after load with 4 of those patients becoming seizure free immediately after administration, with confirmation obtained via EEG (13). Importantly, the group described the need for a therapeutic ideal with properties of ability for rapid dosing with instantaneous onset and minimal toxicity, sedation, and interaction with the hypothesis that levetiracetam best fits such a description among commercially available AEDs (13).

Spencer et al. (14) pointed out the benefit of levetiracetam reaching peak steady state concentrations within an hour of administration; though with the caution that it has demonstrated a more rapid administration rate in a small subset of critically ill patients (14). Lyseng-Williamson (15) also described the clinical tolerability of levetiracetam, noting in her review that adverse effects of major concern in a critically ill patient, such as hypotension, arrhythmias, and cutaneous and hypersensitivity reactions, were not commonalities in the use of levetiracetam in its initial clinical trials (15). Instead, asthenia and somnolence were noted among its major side effects (15).

Nau et al. (16) served to further this data with a retrospective analysis of 51 patients, 18 of whom received a loading dose of 1500 mg levetiracetam. Seventeen of those 18 patients received levetiracetam as first-line monotherapy (16). Of these patients, no drug interactions, cardiac arrhythmias, or adverse hemodynamic events were recorded, similarly suggesting that levetiracetam has the potential to be well tolerated in the critically ill (16). In the same vein, Rösche et al. (17) provided a comprehensive review of literature concerning the treatment of status epilepticus with levetiracetam, though not specifically as a first-line agent. The authors found an on-the-whole rate of levetiracetam terminating status in 53.7–58.1% of patients with the most frequent side effects of irritability and sedation (17).

## Practical Applications in the Current Therapeutic Sequence

Liu et al. (18) served to further this concept with a literature based comparison of intravenous valproate sodium with other antiepileptic drugs by completing meta-analysis performed on five randomized control trials (18). No substantial difference was noted between valproate sodium and levetiracetam in time taken to control status, and similar responses for phenytoin and sodium valproate were found. These are suggestive of similar efficacies of all three agents (18). Of the three, however, the relatively benign side effect profile of levetiracetam makes it an appealing choice of agent given the agents’ equivalent drug efficacies.

Aiguabella et al. (19) set out to determine the efficacy of intravenous levetiracetam as an add-on agent in the management of status in their observational retrospective study of 40 patients. These patients received initially the traditional regimen of a benzodiazepine followed by phenytoin or valproate (19). Ultimately, seizures were controlled in 57.7% of patients with a mean efficacy time of 14.4 h (19). Of the 40 patients, only 26 ultimately received the traditional regimen followed by levetiracetam given as a third-line agent. Those patients have a levetiracetam efficacy of only 46.1%. Interestingly, when levetiracetam was given early, with minimal or no pretreatment, as was done in the cases of 14 subjects, levetiracetam was found to have an efficacy of 78.5% (19). Ultimately, the authors postulate that the higher efficacy of levetiracetam early in therapy may be attributed to the idea that status epilepticus requiring multiple agents to reach control were likely to be more treatment refractory from the outset (19). That being said, Aiguabella et al.’s (19) work raises the notion that while other agents may also readily control status as an early therapy, levetiracetam may do so with at least equal efficacy with less morbidity.

## Discussion

While clinical studies of the use of levetiracetam are undoubtedly not yet sufficient to dictate large-scale changes to clinical practice, a substantial foundation for future clinical trials has been laid. Given multiple case reports of successful use of levetiracetam as an initial agent in the emergent treatment of status, there is certainly basis for the development of larger scale randomized controlled trials allowing for a direct, standardized comparison between traditional first-line agents and levetiracetam for emergent management of status epilepticus. The clinical utility of levetiracetam is most suggested by Misra et al.’s (8) pilot study as well as retrospective analyses of the use of levetiracetam in critically ill patients with seizures and status (8). Perhaps the greatest potential advantages to levetiracetam are its pharmacologic properties and restricted morbidity via minimal side effect and interaction profiles. Studies indicate that levetiracetam is an attractive option in patients who are unable to be treated with traditional first-line therapies. This may serve as a catalyst for the exploration of levetiracetam as a first-line medication. Although existing clinical evidence is inadequate, the recommendations of the Neurocritical Care Society for the management of status show a potential for an expanding role of levetiracetam in the management of status with its inclusion of recommended drugs that may be selected in the urgent management of status. In the same way, Cook et al. (6) foreshadow a shift in the traditional line of medical decision making in status with a small but not insignificant number of physicians choosing levetiracetam early on in the management of status (6). While a jump as extensive as levetiracetam approaching recognition as a first-line agent is still far beyond what current models and data support, the basis for more in-depth studies for its potential to function specifically as a first-line agent is formed with considerable feasibility by these underlying works.

Such a hypothesis exists that levetiracetam may well fit the description of an ideal agent as outlined by Reugg et al. (13). Lyseng and Spencer further support this notion of levetiracetam as a readily administered and rapidly absorbed drug with low frequency of high-risk adverse effects, and in fact, a benign side effect profile when compared directly to other traditional agents (14, 15). This then portends the argument that levetiracetam could 1 day usurp traditional antiepileptic agents in order of administration in status given the rapidity of its administration, absorption, efficacy noted as an early therapy, and minimal side effect profile. Perhaps, it is worthy of first-line administration, ahead of traditional agents. Accomplishing this cannot be done without further endeavors, however. No current randomized controlled clinical trial of levetiracetam used in the management of acute adult status epilepticus currently exists, though the potential yield is substantial. Aiguabella et al. (19) create an interesting scenario in their brief observation that difficult-to-control status is fundamentally what it purports to be challenging to manage (19). For example, a patient whose seizures prove complex to control will likely require multiple agents with repeated dosing in order to successfully stop the seizures. However, a patient with readily responsive seizures may respond initially to levetiracetam as well as traditional antiepileptic drugs, albeit with less risk of interactions, and side effects. It is worth questioning if adverse effects experienced in patients whose seizures ultimately responded to traditional first-line therapy but then experienced complications or side effects such as respiratory depression, could be avoided by a better-tolerated therapy, namely levetiracetam. In this manner, a wider range of patients could be potentially treated earlier, more safely, and just as effectively as with mainline drugs used in status.

Certainly no pharmacotherapy is without fault, and it remains both possible and likely that levetiracetam carries administration concerns not yet apparent to clinical practitioners. Just as our understanding of its clinical benefits are restricted by limited data, so too is our comprehension of its potential pitfalls. With the growing establishment of levetiracetam’s clinical pedigree, as well as ever-increasing desire for an efficacious, accessible, and comparatively benign agent for the emergent treatment of status, levetiracetam may prove a worthy contender in the future of acute treatment of status epilepticus.

## Conflict of Interest Statement

The authors declare that the research was conducted in the absence of any commercial or financial relationships that could be construed as a potential conflict of interest.
